# An Economic Impact Model for Estimating the Value to Health Systems of a Digital Intervention for Diabetes Primary Care: Development and Usefulness Study

**DOI:** 10.2196/37745

**Published:** 2022-09-26

**Authors:** Brenton Powers, Amy Bucher

**Affiliations:** 1 Lirio Knoxville, TN United States

**Keywords:** return on investment, value, payment model, cost, economic impact, digital health, eHealth, diabetes, primary care, email

## Abstract

**Background:**

Diabetes is associated with significant long-term costs for both patients and health systems. Regular primary care visits aligned with American Diabetes Association guidelines could help mitigate those costs while generating near-term revenue for health systems. Digital interventions prompting primary care visits among unengaged patients could provide significant economic value back to the health system as well as individual patients, but only few economic models have been put forth to understand this value.

**Objective:**

Our objective is to establish a data-based method to estimate the economic impact to a health system of interventions promoting primary care visits for people with diabetes who have been historically unengaged with their care. The model was built with a focus on a specific digital health intervention, Precision Nudging, but can be used to quantify the value of other interventions driving primary care usage among patients with diabetes.

**Methods:**

We developed an economic model to estimate the financial value of a primary care visit of a patient with diabetes to the health system. This model requires segmenting patients with diabetes according to their level of blood sugar control as measured by their most recent hemoglobin A_1c_ value to understand how frequently they should be visiting a primary care provider. The model also accounts for the payer mix among the population with diabetes, documenting the percentage of insurance coverage through a commercial plan, Medicare, or Medicaid, as these influence the reimbursement rates for the services. Then, the model takes into consideration the population base rates of comorbid conditions for patients with diabetes and the associated current procedural terminology codes to understand what a provider can bill as well as the expected inpatient revenue from a subset of patients likely to require hospitalization based on the national hospitalization rates for people with diabetes. Physician reimbursement is subtracted from the total. Finally, the model also accounts for the level of patient engagement with the intervention to ensure a realistic estimate of the impact.

**Results:**

We present a model to prospectively estimate the economic impact of a digital health intervention to encourage patients with documented diabetes diagnoses to attend primary care visits. The model leverages both publicly available and health system data to calculate the per appointment value (revenue) to the health system. The model offers a method to understand and test the financial impact of Precision Nudging or other primary care–focused diabetes interventions inclusive of costs driven by comorbid conditions.

**Conclusions:**

The proposed economic model can help health systems understand and evaluate the estimated economic benefits of interventions focused on primary care and prevention for patients with diabetes as well as help intervention developers determine pricing for their product.

## Introduction

The Centers for Disease Control and Prevention (CDC) estimates that in 2018, 34.2 million Americans or 10.5% of the US population had diabetes [1]. The economic costs associated with diabetes in the United States are estimated to be in excess of US $327 billion total, with US $237 billion coming from direct medical costs and the rest in productivity reductions. For individual patients, the economic impact of diabetes can be catastrophic, with estimated annual medical expenses of US $9600 directly attributable to diabetes and a total of US $16,750 on average, with medical expenditures exceeding 2.3 times the amount incurred by patients without diabetes [2]. People with diabetes are also likely to have comorbid conditions that contribute significantly to their medical expenses [3,4], with 40% of adults with diabetes having at least 3 comorbid chronic diseases [5]. Moreover, people with diabetes have an increased odds of inpatient admission [6], with 34% of patient admissions occurring among people with diabetes [1] and a higher likelihood of readmission within 30 days of discharge [7] compared to people without diabetes (20.5%) [8]. In short, diabetes is both prevalent and expensive.

It is also common for people with diabetes to not be aware that they have the condition, which limits their ability to engage in appropriate care. In 2018, 21.4% of US adults with diabetes did not report having the condition—a total of 7.3 million people whose laboratory results qualify for a diagnosis [1]. This group is almost certainly not engaged in recommended condition management behaviors such as primary care and specialist appointments specifically to address diabetes. Engagement is lacking in people who are aware of their diabetes status as well; for example, one study on people with diabetes found 16.2% were no-shows to their last scheduled primary care appointment [9]. In general, missed medical appointments are estimated to cost the US health care system in excess of US $150 billion per year [10], indicating a need for interventions that increase patient attendance.

Noncompliance with recommended care has serious consequences for both individuals and systems. A comparison of people with diabetes who were compliant and noncompliant with American Diabetes Association recommendations for primary care found significant improvements in medical utilization, including a reduction in the need for inpatient care [11] when recommendations were followed. For example, one study found that promoting lifestyle changes around diet and physical activity for people with diabetes and prediabetes yielded significant cost savings related to medical care over a 10-year time period [12]. Regular well visits with providers are also associated with positive outcomes for people with diabetes. Research suggests that about 30% of patients working with a primary care provider (PCP) achieve hemoglobin A_1c_ (HbA_1c_) control by 1 year [13], and in general, interventions targeting appointment attendance are associated with better diabetes outcomes [14].

Naturally, many behavioral interventions for people with diabetes focus on adherence to recommended clinical pathways to improve outcomes. In the digital health space, these interventions often focus on blood glucose monitoring, diabetes education, and lifestyle modifications [15] as the key modifiable behaviors. In general, these digital health apps have heterogeneous outcomes with some promise to help lower HbA_1c_ levels and other biometrics as well as improve patient self-efficacy and condition management skills [16]. For example, a meta-analysis and evidence review of app-based interventions for type 2 diabetes suggests an overall reduction in HbA_1c_ levels among users, particularly among younger users, and when there is a feedback loop involving the provider [17]. A number of these digital health interventions claim to reduce the system- and individual-level costs of diabetes. For example, in one longitudinal claims-based study of Omada Diabetes Prevention Program, participants incurred reduced health care costs at 1 year after enrollment, including reduced inpatient costs [18]. Evidence suggests that digital health interventions may be an effective tool to augment clinical-based diabetes care, but there remain gaps in the evidence base, particularly around the economic impact [19,20] and a relative dearth of interventions focused on supporting traditional clinical pathways such as primary care. Finally, models focused on cost reduction may overlook the value to health systems from revenue generation via primary care utilization.

In short, diabetes is a complex condition that can drive costs from a number of sources, whether through more intensive preventative care needs, frequently comorbid conditions, or complications or sequelae requiring more expensive treatment. Given the focus of most diabetes digital health interventions, estimates of the value of these interventions often focus on the impact of lifestyle changes rather than appropriate utilization of preventative care such as regular PCP appointments. Primary prevention and regular care are likely to deliver value to the health system beyond their direct impact and have been considered as components of a value-based agreement for diabetes care [21,22]. We believe there is cause to focus on primary care and well visits as a modifiable behavior for people with diabetes and a need to quantify the economic value of doing so in order to appropriately prioritize interventions.

In this paper, we put forth a conceptual model and process to prospectively estimate the downstream economic benefits of a specific primary care–focused diabetes intervention, Precision Nudging for diabetes, to a health system. This model will ultimately provide the basis for assessing the intervention’s value postimplementation, with a focus on revenue to the health system rather than a specific clinical outcome or quality-adjusted life years [23]. Although the model was developed to assess a specific intervention, we believe it is generalizable to understand the economic impact of other digital health initiatives focused on primary care utilization for people with diabetes. This would permit value-based pricing of commercial digital health interventions as well as an evidence-based method for health systems and provider organizations to determine whether and how to utilize such interventions for their populations.

## Methods

### Overview

The model was developed prospectively to quantify the impact of a specific diabetes behavioral intervention called as Precision Nudging. The key outcome measure for the intervention is the number of patients who attend a PCP appointment; therefore, the model focuses on assigning a financial value to each appointment to understand the financial impact of the intervention. The model focuses on the value that accrues to a health system, taking into account direct and indirect costs of diabetes as well as provider reimbursements, and was specifically intended to help health systems evaluate the value of Precision Nudging for their population.

### Precision Nudging Intervention

Precision Nudging for diabetes is an English-language messaging intervention for people with a diagnosis of type 1 or type 2 diabetes, focused on the target behaviors of scheduling and attending a well visit with a PCP either once, twice, or 4 times per year based on their most recent A_1c_ value as recommended by the Healthcare Effectiveness Data and Information Set (HEDIS) [24]. A behavioral reinforcement learning algorithm [25-27] selects behavioral science–based message content to send to eligible patients to prompt them to schedule and attend a diabetes well visit. The algorithm is designed to select messages based on recipient characteristics with an emphasis on prior recipient behavioral responses (ie, message opens and clicks and appointment scheduling and attendance), to maximize the likelihood the message is opened and the call to action heeded.

The messages are designed to address specific barriers people might have to scheduling and attending a diabetes well visit, identified through primary research during the intervention development process and a comprehensive literature review. Behavioral designers use an intervention mapping process to categorize the determinants and align them with behavior change techniques [28,29], which are then operationalized as message content (subject lines and body copy) and visual designs. The behavioral reinforcement learning algorithm compiles a complete message for each recipient from 10 subject lines and 26 body copy/visual design options, for a total of 320 unique message combinations that a patient might receive. Eligible patients receive 1 email per week for 5 weeks, followed by an 8-week pause, and then another 1 message per week for 5 weeks. This pattern repeats until the patient either unsubscribes from the intervention or takes action by scheduling and attending a PCP visit. The key outcome metric associated with Precision Nudging is the completion of a primary care appointment. The outcome of appointment attendance serves as the basis for the economic model.

### Establishment of Diabetes Impact to the Health System

A best practice in developing economic impact models is to clearly identify the entity to which the value is delivered [30]. In this case, it is the health system. The first step in the economic model is to establish the impact of diabetes to the health system with respect to diabetes severity and risk level, as characterized by patients’ most recent HbA_1c_ value. This analysis will vary by health system depending on their patient population and payer mix. First, an understanding of the health system’s patient population with diabetes must be established. This occurs by parsing electronic medical record data to identify patients with a documented diagnosis of diabetes (excluding gestational or medication-induced diabetes). The method of documenting a diabetes diagnosis may vary depending on system and implementation. In our case example, the health system maintained a diabetes registry within its Epic implementation that provided the base estimates for diabetes in the patient population.

From there, we segmented patients with diabetes based on their last recorded HbA_1c_ value. Following Healthcare Effectiveness Data and Information Set measures [24], patients were grouped as well-controlled if their HbA_1c_ level was below 7, moderately controlled if their HbA_1c_ level was between 7 and 9, and uncontrolled if their HbA_1c_ level was above 9. Patients who did not have an HbA_1c_ value in their records were classified as moderately well-controlled for messaging purposes, with the rationale that their PCP would order A_1c_ testing in a first or second visit and then the patient would be classified based on their actual value. In addition to facilitating guideline-consistent recommendations for care within the intervention, segmenting patients by HbA_1c_ value also permits the risk adjustment that has been identified as a best practice for understanding the value [31].

Because the intervention targets patients who are not up-to-date with recommended PCP appointments, we also set eligibility parameters based on the date of the last primary care visit. The recommended frequency of primary care visits differs by HbA_1c_ level; therefore, well-controlled patients are recommended 1 visit per year, moderately well-controlled 2 visits per year, and poorly controlled 4 visits per year, consistent with American Diabetes Association guidelines [32]. We classify patients who have not had a primary care visit in the appropriate lookback period and do not have one scheduled in the next 3 months as unengaged and consider them eligible for intervention messaging.

In working with a health system, it may be necessary to repeat the exercise of identifying patients with diabetes and classifying them by HbA_1c_ level per market or care site where the intervention will be offered, particularly if the payer mix or provider reimbursements vary by location. Table 1 offers an example of what this documentation may look like per location.

**Table 1:** Quantification of the population with diabetes within a health system or market by the hemoglobin A_1c_ value and being unengaged with care due to not having a past appointment within the recommended time frame or a future visit scheduled.

Patient HbA_1c_^a^ level	Criterion 1 (HbA_1c_ value)	Patients (n)	And criterion 2 (appointment overdue)	Patients (n)	And criterion 3 (next appointment more than x time in future)	Total unengaged population
Controlled	HbA_1c_<7	XXX	Last appointment >11 months	XXX	3 months	XXX
Moderately controlled	HbA_1c_ ≥7 to <9	XXX	Last appointment >5 months	XXX	3 months	XXX
Uncontrolled	HbA_1c_≥9	XXX	Last appointment >2 months	XXX	3 months	XXX

^a^HbA_1c_: hemoglobin A_1c_.

### Identification of Current Procedural Terminology Codes

The next step in developing the model was to identify the current procedural terminology (CPT) codes corresponding to the procedures that may take place during a primary care visit for a patient with diabetes. Then, each code was assigned an allocation based on the percentage of patients who would be expected to receive that code on a given visit. For example, every PCP visit merits a CPT code for physician office visit; therefore, that code receives a 100% allocation, while the CPT code for a lipid panel is assigned a 44% allocation based on the national rate of hypercholesterolemia among people with diabetes [1]. A total of 10 CPT codes were identified and assigned an allocation percentage (see Table 2). The identified codes include professional services only that can be bundled within the parameters of a physician visit. Hospital laboratory services related to the visit were excluded.

CPT code volumes were then adjusted based on the percentage of patients in the health system with a status of well controlled, moderately controlled, or uncontrolled and the corresponding number of annual wellness visits recommended (1, 2, or 4, respectively). Finally, the health system’s patient population was characterized in terms of its payer mix to assign a financial value to each CPT code and its projected frequency. Dollar amounts were assigned based on typical reimbursement rates for each CPT code by plan, as determined by (1) Medicare: based on data from Palmetto GBA, (2) Medicaid: based on the rates for the state where the health system is located, and (3) commercial health plan: based on an average 135% of the Medicare reimbursement.

**Table 2:** The 10 current procedural terminology codes and their corresponding allocations based on the percentage of patients with diabetes likely to need them in a primary care provider appointment in order to determine the revenue potential of each appointment.

Current procedural terminology code description	Allocation (%)
Physician office visit	100
Hemoglobin A_1c_ level	100
Urinalysis	100
Lipid panel	44
Complete blood count with auto-differential	100
Education on self-managed blood pressure setup	68
Education on self-managed blood pressure monitor	68
Tobacco cessation	22
Diabetic foot examination	100
Depression	25

### Physician Reimbursement

The revenue to the professional practice is offset by provider reimbursement. Physicians are reimbursed based on the CPT codes they submit, with each code having a relative value unit assigned to it by the Centers for Medicare and Medicaid Services (CMS). The worked relative value units are multiplied by a conversion factor, which may include a geographical adjustment, to arrive at a dollar value for each service. In order to account for physician reimbursement in the model, provider reimbursement was estimated using the median Medical Group Management Association conversion factor on CMS relative value units for the CPT codes charged [33].

### Inpatient Care Reimbursement

People with diabetes are more likely to be admitted to inpatient care than people without diabetes [6]; the CDC reports that 339 out of 1000 people with diabetes may experience the need for inpatient care over a 1-year period [1]. Fortunately, regular PCP visits may reduce the risk of inpatient care, as following American Diabetes Association guidelines is associated with decreased admission rates [11]. Because the Precision Nudging intervention focused on the unengaged population who were out of compliance with the recommended cadence of primary care visits, we focused on only that group in calculating potential inpatient care costs. Revenue is calculated on the margin from reimbursement by commercial health plans, Medicare, and Medicaid in the proportion those payers cover the unengaged patients exist in the system’s population. These calculations yield a value summary.

### Summary Calculation

When applied to the population of patients with diabetes within a health system, this economic impact model yields an annual incremental value summary for the intervention—that is, a specific dollar value per primary care visit scheduled as a result of the intervention. The total margin is calculated by adding total professional practice revenue and total hospital margin and subtracting total provider compensation. This amount can be annualized and divided by the number of visits attended to arrive at a value per visit. This dollar amount supports the calculation of a return on investment based on the costs of implementation and operational support for the intervention.

The final calculation using the economic impact model with the inputs described above is as follows:

Professional practice revenue (annual) + inpatient revenue (annual) – provider compensation (annual) = Total annual reimbursement to health system / Total attended PCP appointments from 100,000 patients = Dollar value per appointment

### Ethical Considerations

The development of the economic impact model did not utilize human subjects and so was not submitted for institutional review board review.

## Results

Although we are unable to provide the specific calculation used with the health system implementation due to its use of proprietary information, the following example illustrates the process with a resulting dollar value per visit. The example is based on a (hypothetical) population of 100,000 patients with diabetes diagnoses; segment breakdowns are modeled on national averages where available.

### Diabetes Segments in the Patient Population

According to the 2020 CDC National Diabetes Statistics Report, 50% of adults with diabetes had A_1c_ values below 7, 35.5% had A_1c_ values between 7 and 9, and 14.5% had A_1c_ values above 9 [1]. We use this breakdown in our sample population of 100,000 patients. Next, given a lack of national data on engagement with primary care for patients with diabetes by level of glycemic control, we look at the actual health system where the intervention was deployed for engagement rates in each A_1c_ category. Within those segments, 13.1% (6550/50,000) of the patients with A_1c_ values below 7 were overdue for their PCP visit, as were 29.8% (10,579/35,500) of the patients with A_1c_ values between 7 and 9, and 61.1% (8860/14,500) of the patients with A_1c_ values above 9. Finally, 96.2% (6301/6550) of the overdue patients with A_1c_ values below 7 did not have a future PCP visit scheduled, nor did 86.7% (9172/10,579) of the patients with A_1c_ values between 7 and 9, nor 75.6% (6698/8860) of the patients with A_1c_ values above 9. The number of patients from the original population of 100,000 considered unengaged with their diabetes care is 22,171 people. The calculation of the unengaged sample eligible for the intervention is summarized in Table 3.

**Table 3:** Values based on the health system where the intervention was used to describe a population of 100,000 patients with diabetes by hemoglobin A_1c_ value who do not have a past appointment within the recommended time frame or a future visit scheduled and would therefore be eligible for the intervention.

Patient HbA_1c_^a^ level	Criterion 1 (HbA_1c_ value)	Patients (N=100,000)	And criterion 2 (appointment overdue)	Patients (n=25,989)	And criterion 3 (next appointment more than x time in future)	Total unengaged population (eligible for messages) (n=22,171)
Controlled	HbA_1c_<7	50,000	Last appointment >11 months	6550	3 months	6301
Moderately controlled	HbA_1c_≥7 to <9	35,500	Last appointment >5 months	10,579	3 months	9172
Uncontrolled	HbA_1c_≥9	14,500	Last appointment >2 months	8860	3 months	6698

^a^HbA_1c_: hemoglobin A_1c_.

### Identification of CPT Codes

The national hospital data in the United States showed that approximately 67% of the patients in 2022 had commercial health insurance or were self-paying, while 20.5% had Medicare and 13.2% had Medicaid [34]. We rounded commercial health insurance coverage (which is the most lucrative for health systems) down to 66.3% in order to arrive at a total of 100%.

Applying that breakdown to the hypothetical population of 22,171 patients eligible for the intervention by A_1c_ level allows us to look at the CPT codes each patient may be charged each year if they participate in recommended diabetes care. Then, prices from the Palmetto GBA and state Medicaid reimbursement rates are applied to calculate the dollar value associated with the recommended care for the eligible population. Because the frequency of the recommended diabetes visits varies by the A_1c_ level, this exercise should be done separately for each A_1c_ category. For illustrative purposes, the CPT code–based revenue potential of a diabetes well visit for the uncontrolled group (A_1c_>9) is described in Table 4.

Please note that the calculations for the moderately controlled and well-controlled groups are not included here but are part of the actual analysis. For this example, the total potential annualized revenue to the health system across all 3 A_1c_ categories comes to US $44,778,974. Considering only the unengaged patients in the sample, the total potential annual reimbursement to the health system is US $1,313,177.

**Table 4:** Potential annualized revenue from an eligible patient population with hemoglobin A_1c_ levels above 9 (uncontrolled group) based on the payer mix and the expected current procedural terminology codes that could be billed during a primary care provider visit (N=14,500).

CPT^a^ code description	Commercial plan		Medicare		Medicaid	
	Code allocation (n=9614), n (%)	Reimbursement (USD) (Total=US $8,986,351)	Code allocation (n=2973), n (%)	Reimbursement (USD) (Total=US $1,852,541)	Code allocation (n=1914), n (%)	Reimbursement (USD) (Total=US $670,742)
Physician office visit	9614 (100)	5,669,134	2973 (100)	1,225,946	1914 (100)	506,368
Hemoglobin A_1c_	9614 (100)	743,081	2973 (100)	115,471	1914 (100)	68,827
Urinalysis	9614 (100)	N/A^b^ (bundled with physician office visit)	2973 (100)	N/A (bundled with physician office visit)	1914 (100)	N/A (bundled with physician office visit)
Lipid panel	4230 (43.9)	450,869	1308 (43.9)	70,063	842 (43.9)	41,771
Complete blood count with auto-differential	9614 (100)	297,309	2973 (100)	46,200	1914 (100)	27,523
Education on self-managed blood pressure setup	6538 (68)	92,552	2022 (68)	20,014	1302 (68)	10,712
Education on self-managed blood pressure monitor	6538 (68)	1,585,158	2022 (68)	342,789	1302 (68)	N/A
Tobacco cessation	2115 (21.9)	87,894	654 (21.9)	19,007	421 (21.9)	7942
Diabetic foot examination	9614 (100)	N/A (bundled with physician office visit)	2973 (100)	N/A (bundled with physician office visit)	1914 (100)	N/A (bundled with physician office visit)
Depression	2404 (25)	60,354	743 (24.9)	13,051	479 (25)	7599

^a^CPT: current procedural terminology.

^b^N/A: not applicable.

### Physician Reimbursement

Next, we calculated the expected physician reimbursement based on the CPT codes they would be able to submit for the intervention population. This provider compensation, described in Table 5, will be subtracted from the practice revenue. At this point, we assume that not all patients targeted by the intervention will participate in a PCP appointment; based on typical engagement rates for Lirio’s digital health interventions, we chose a conservative estimate of 10% appointment attendance among the total unengaged population (n=2235).

Multiplying the 5006.87 relative value units that providers can submit by the Medical Group Management Association conversion factor of US $41.94 yields a monthly provider reimbursement of US $17,499 or US $209,988 per year.

**Table 5:** An estimate for provider reimbursement based on current procedural terminology codes submitted during a primary care provider appointment as predicted by comorbidity rates with diabetes.

Current procedural terminology code description	Allocation (n=2217), n (%)	Total with relative value units (n=5006.87)
Physician office visit	2217 (100)	4256.64
Hemoglobin A_1c_ level	2217 (100)	N/A^a^
Urinalysis	2217 (100)	N/A
Lipid panel	975 (43.9)	N/A
Complete blood count with auto-differential	2217 (100)	N/A
Education on self-managed blood pressure setup	1508 (68)	271.36
Education on self-managed blood pressure monitor	1508 (68)	361.81
Tobacco cessation	488 (22)	117.06
Diabetic foot examination	2217 (100)	N/A
Depression	554 (24.9)	N/A

^a^N/A: not applicable.

### Inpatient Care Reimbursement

Using the 339 in 1000 rate of hospitalization among patients with diabetes [1] and considering the 10% of the unengaged population (n=2235) that we predict to capture in the intervention, we estimate that approximately 757 patients will receive inpatient care over the next year. In our actual health system implementation, expected reimbursement per patient by payer type for an inpatient stay was provided. For purposes of the modeling exercise, we used the following values: (1) Medicare: US $2000 based on the CMS Financial Year 2023 national adjusted operating standardized amount, non–labor-related costs [35]; (2) Medicaid: US $1280 or 64% of the Medicare reimbursement (proportional to the reimbursement for physician office visit); and (3) commercial health plan: US $2700 or 135% of the Medicare reimbursement. Assuming an even mix of hospitalization across payer type and A_1c_ level and using these reimbursement values, we calculated an annual value summary from inpatient care of US $1,796,506.

### Summary Calculation

Using the inputs described above, the final calculation using the economic impact model is as follows:

Professional practice revenue (annual, US $1,313,177) + inpatient revenue (annual, US $1,796,506) – provider compensation (annual, US $209,988) = Total annual reimbursement to health system (US $3,050,957) / Total attended PCP appointments from 100,000 patients (2217) = Dollar value per appointment (US $1297)

The final value calculated in this example was US $1297 per PCP appointment attended by an unengaged patient with diabetes. This value can now become the basis for pricing discussions and return on investment calculations.

## Discussion

### Principal Findings

This economic impact analysis provides a method for estimating the immediate and downstream value to a health system of the Precision Nudging intervention targeting primary care for patients with diabetes. This model could also be applied to estimate the value for other interventions focused on connecting patients with diabetes with primary care or adapted to use for other patient populations. Being able to forecast the total economic value of an intervention is a critical prerequisite for widespread adoption [36] and will help both intervention designers and health system customers more quickly identify which tools are effective [37]. Importantly, the model takes into account the different patient risk profiles associated with various levels of glycemic control as measured by HbA_1c_ levels. It also relies heavily on publicly available data from CMS, the CDC, and similar organizations, making it possible for other stakeholders to adapt this model to assign a value to their own interventions.

This model was developed as a pricing exercise ahead of implementing an intervention. An immediate opportunity is to populate the model with actual health system data and determine whether its predictions align with real-world performance. Precision Nudging for diabetes is currently used in the health system for whom this model was developed. Given the model’s 1-year time horizon, patient claims data can be used 12 months postimplementation to verify whether the estimated impacts were realized and how the model may need to be corrected to more accurately reflect outcomes.

We anticipate the model will need to be adjusted as we learn more about the uptake and effect of interventions targeting primary care use. For example, not all patients who are targeted for a digital health intervention will take action as a result. Although in one study, 65% of adults with diabetes expressed willingness to use a digital health tool to manage their diabetes even if it had a minimal effect on their outcomes [38], the intention-behavior gap is well-documented [39] and it is well-known that digital health adoption in general lags expectations. Therefore, we recommend adjusting any economic impact estimates to reflect a portion of the population that may take action, especially if using the model as part of a pricing or sales exercise. The conservative adoption value of 10% we used in the initial model will likely need to be edited to reflect actual engagement rates.

In fact, it is likely that a validation study will find greater uptake than the 10% estimate used to build the model. This is because the particular digital health intervention being studied meets best practices for adoption [36], such as being easy for patients to use and providing a self-evident clinical benefit (adherence to recommended provider visits). Product teams also constantly iterate and improve on digital health offerings, ostensibly improving their uptake and impact. Over time, it may be possible to quantify intervention design factors that influence uptake and use them as inputs to an economic impact model like this one. Relatedly, it will be important to reassess the intervention’s impact over time as improvements are made to the intervention and how it is implemented.

Another opportunity area is to expand this type of economic modeling to digital health interventions that promote provider appointments for other chronic conditions, especially conditions where patients often have multiple comorbidities. There are likely significant downstream cost benefits to adequate primary care for patients with these conditions, and better understanding the nature of those benefits can help support health system choices on where to focus their patient support efforts.

We believe this kind of economic impact analysis will help determine the appropriate role of digital health interventions in value-based care contracts, which are increasingly common in the United States [21]. This model, along with research on leveraging primary care relationships for the care of chronic conditions, suggests that there is significant immediate and downstream value to using scalable technology to connect patients to their PCPs, such as reduced cost compared to specialist care [40], reduced mortality [41], and improved outcomes [42].

Finally, we recognize that the economic value of an intervention to a health system is only part of the story. To be successful over time, interventions must also support patient quality of life [43]; indeed, economic models focused on quantifying value to entities such as governments, employers, or payers often include quality-adjusted life years or productivity-based outcomes [23]. Although this economic value model is health system revenue–focused, it accounts for factors that matter to the patient experience. Avoiding progression of diabetes and related comorbidities and otherwise maintaining a better quality of health should positively impact patient quality of life. Ultimately, the goal is not to drive more health care utilization but rather to drive appropriate health care utilization. A future research direction is to understand patient experience consequent to interventions like Precision Nudging and ensure that the interventions deliver improvements for patients as well as systems.

### Limitations

A major limitation that undergirds the need to pressure test the model with real world data is that it is unlikely any health system will perfectly mirror the publicly available data used to build the model. For example, we used average comorbidity rates of other health conditions for people with diabetes to estimate how frequently providers would be able to charge specific CPT codes. It is very likely that within any given health system, actual patient comorbidity rates differ from those averages. Given that part of the intent of the model is to guide pricing around primary care interventions, there is a limited acceptable margin of error for differences between the estimates and actual data. If the model is overly generous in its value calculations, it will not be accepted by health systems as a pricing tool. It also requires some effort for any health system to populate the model with their own data (eg, their patient payer mix), and it is likely some organizations will prefer not to go through the exercise. There is a tension between using average or typical data to ballpark the value of an intervention and the labor required to arrive at a more precise estimate by using actual health system data.

The inclusion of inpatient care as a value-add in the model is also a concern because it is at odds with the goals of limiting patient costs and improving quality of life. In an ideal state, primary care would help stave off hospitalizations rather than prompting them. Unfortunately, in working with unengaged patient populations, it is likely that some of them will require hospitalization subsequent to a primary visit due to unaddressed health issues. If interventions like Precision Nudging that target more regular and appropriate use of primary care are successful, over time, we hope that the value from hospitalizations in this model will need to be reduced. Finally, it is a limitation that we are unable to share the specific data used in the initial development of the model for a real health system. We have used mock data in our results that provide a similar output and offer a reasonable ballpark dollar value for the diabetes primary care visit.

### Conclusion

For digital health interventions targeting primary care to receive greater attention and be implemented in health systems, it is important to quantify the value they deliver. To our knowledge, there have been no widely accepted ways to value the economic impact of interventions that encourage appropriate use of primary care among people with diabetes. This paper offers a model based largely on publicly available data that would allow the calculation of a dollar value for a primary care visit for a patient with diabetes; this facilitates intervention pricing by vendors as well as prioritization by health systems or other customers as they consider the mix of services they use to close gaps in patient care. So much focus in digital health interventions for diabetes has been around blood glucose monitoring, education and lifestyle change, but appropriate use of primary care is a powerful tool too. Use of this model should help ensure that primary care–focused interventions receive their due recognition as effective tools to treat people with diabetes and prevent the progression of illness and its comorbidities.

## Abbreviations

CDC: Centers for Disease Control and Prevention
CMS: Centers for Medicare and Medicaid Services
CPT: current procedural terminology
HbA_1c_: hemoglobin A_1c_
PCP: primary care provider
